# Fabrication and Enhanced Performance Evaluation of TiO_2_@Zn/Al-LDH for DSSC Application: The Influence of Post-Processing Temperature

**DOI:** 10.3390/nano14110920

**Published:** 2024-05-24

**Authors:** Altaf Hussain Rajpar, Mohamed Bashir Ali Bashir, Ethar Yahya Salih, Emad M. Ahmed

**Affiliations:** 1 Department of Mechanical Engineering, College of Engineering, Jouf University, Sakaka 72388, Saudi Arabia; ahrajpar@ju.edu.sa; 2 Department of Renewable Energy, College of Renewable Energy and Environmental Sciences, Al-Karkh University of Science, Baghdad 10081, Iraq; ethar988@gmail.com; 3 Department of Electrical Engineering, College of Engineering, Jouf University, Sakaka 72388, Saudi Arabia

**Keywords:** TiO_2_, dye-sensitized solar cell, layered double hydroxide, mixed metal oxide

## Abstract

A sequence of dye-sensitized solar cells is proposed, utilizing TiO_2_@Zn/Al-layered double hydroxide (LDH) as their starting materials, in which Ruthenizer N719 was used as a photon absorber. The anticipated system was turned into sheet-like TiO_2_@mixed metal oxide (MMO) via post-processing treatment. The crystal quality indicated a relation to power conversion efficiency (PCE); this was combined with a comparable morphology profile. In detail, the optimum DSSC device exhibited average sheet-like thickness and a dye loading amount of 43.11 nm and 4.28 $\times 10^{-3}$ mM/cm^−2^, respectively. Concurrently, a considerable PCE enhancement of the optimum DSSC device (TiO_2_@MMO-550°) was attained compared to pristine MMO (0.91%), which could be due to boosted electron transfer efficiency. Of the fabricated devices, DSSC fabricated at 550° exhibited the highest PCE (1.91%), with a 35.6% enhancement compared to that obtained at 450°, as a result of its increased open-circuit voltage (3.29 mA/cm^2^) and short-circuit current (0.81 V). The proposed work delivers an enhanced efficiency as compared to similar geometries.

## 1. Introduction

The increased demands for petroleum and fossil fuels as the main source of energy, as well as the environmental awareness of their uses, are continuing to exert pressure on the already-existing world energy infrastructure. Therefore, a suitable replacement for sunlight conversion into electrical energy is of great need [1,2,3]. The technology of dye-sensitized solar cells (DSSCs) has attracted substantial consideration among the research and industrial societies because of its encouraging potential for relatively low production cost and high outcome efficiency [4,5,6,7,8]. However, the need for easy production and cost-effectiveness techniques of semiconductor photo-electrodes is of crucial significance when considering DSSC fabrication [9]. Herein, the utilization of mixed metal oxide (MMO) acquired via the calcination of layered double hydroxide (LDH) has demonstrated promising behavior as a photo-anode material for DSSC application; this is mainly attributed to the tunable composition, high stability, large surface area, and relatively low production cost as well as forthright preparation approaches [10,11,12]. Herein, LDH is utilized in variety of applications, particularly in the optoelectronic field [13,14]. Interestingly, the calcination of the addressed structure results in LDH’s interlayer collapse, after which a variety of MMO structures can be attained, depending on the divalent and trivalent metal ions employed [15,16]. MMOs have revealed significant interest for a diverse field of applications, such as super capacitors, gas sensors, photodetectors, ultraviolet and visible light photo-catalysts, etc. For the fabrication of DSSCs, MMOs are used as an active photo-electrode material due to a high electron injection efficiency, a high specific area for dye loading, rapid photo-response behavior, and a comparable energy band gap to TiO_2_ and ZnO [17,18,19,20]. 

Several reports demonstrated the utilization of MMO-based Zn/Al-LDH as a precursor in DSSCs applications, through which a particular emphasis was placed on the active part of ZnO in the MMO matrix [10,21,22,23]. Moreover, the addition of metal and metal oxide nanoparticles, such as G, Cu, GO, CuO, and TiO_2_, to the MMO matrix, was also investigated [24,25,26,27,28]. Herein, the proposed study delivers an investigation concerning the addition of TiO_2_ to the matrix of MMO-based Zn/Al-LDH. Furthermore, the effect of post-processing temperature was also systematically deliberated, where an enhancement of 35.6% was acquired in the PCE profile at 550° (1.91) compared to that at 450° (1.41). Furthermore, an addition of TiO_2_ to the utilized MMO anode materials with cautious control of the post-processing temperature showed a 109.9% increase in the overall output efficiency. The attained efficiency was found to be higher than other outcomes, in similar geometries.

## 2. Materials and Methods

### 2.1. Preparation of TiO_2_ and TiO_2_@LDH

TiO_2_ nanoparticles were synthesized via a typical hydrothermal process. Particularly, titanium (IV) *iso*-propoxide, as a precursor, and ethanol were mixed with a ratio of 10/100 under a constant stirring rate of 800 rpm for 30 min. The resultant mixture was then autoclaved under 200 °C for 18 h. The achieved mixture was multi-washed and dried in an air oven at 65 °C. Hereinafter, the obtained powder was annealed at 450 °C for 1 h in a furnace. Concurrently, TiO_2_@Zn(Al)O-MMO utilizing Zn^2+^/Al^3+^-LDH as a starting material was produced using both a co-precipitation method as well as a thermal treatment temperature approach. Typically, Zn(NO_3_)_2_·6H_2_O and Al(NO_3_)_2_·9H_2_O were mixed with a ratio of 8:1 in 150 mL of distilled water at 27 °C, with a continuous stirring rate of 600 rpm for 30 min; a precise homogenous growth was attained via the addition of NaOH (1.25 M), wherein a pH of 7.5 was obtained. Subsequently, TiO_2_ (0.1 gm) was added to the Zn/Al-LDH solution and left to stir for 2 h, after which the resultant solution was multi-washed and centrifuged to remove any undesired impurities. The resultant paste was diluted using a few drops of EtOH and then transferred to a freshly washed fluorine-doped tin oxide (FTO) substrate via a three-cycles spin coating technique, with an area of 1 cm^2^; the thin film was annealed at 150° for 10 min after each deposition. The attained film/s were treated at a post-processing temperature of 450 °C, 550 °C, and 650 °C for 1 h with a heating rate of 5 °C/min in a muffle furnace; the films were designated as TiO_2_@MMO-T, with T signposts to indicate the employed post-processing temperatures. 

### 2.2. Fabrication of DSSC

Herein, 5 mM of dye N719 (535-bisTBA Ruthenizer, Solaronix, Aubonne, Switzerland) was adsorbed by the attained MMO-550° and TiO_2_@MMO-T layers via immersion for a duration of 3 h at room temperature. Synchronously, the Pt electrode was acquired using a DC sputtering technique on the FTO glass, and later sandwiched with the fabricated photo-anode with polymer film (100 μm) as a spacer. Next, Iodolyte (iodide/triiodide, Z50, Solaronix, Aubonne, Switzerland) was inserted between the photo-anode and the Pt electrode through capillarity.

### 2.3. Characterizations

The thermal properties of the LDH layer were investigated using thermogravimetric and differential thermal approaches (SBTA851-Mettler Toledo, TGA/DTG, Columbus, OH, USA), while the structural characteristics of the fabricated films were recorded using an X-ray diffractometer (XRD, AXS D8, Bruker, Billerica, MA, USA) under CuKα radiation and a 1.54 nm wavelength. Furthermore, the surface morphologies of the deposited films were inspected using field emission scanning electron microscopy (SU8030, FE-SEM, HITACHI, Tokyo, Japan). The optical analyses were conducted using two different approaches, namely, photoluminescence (PL, LS-50B, PerkinElmer, Waltham, MA, USA, excitation wavelength of 350 nm) and ultraviolet visible light (UV–Vis, Shimadzu, Kyoto, Japan) spectroscopies. The photovoltaic characteristics behavior of the fabricated DSSC was assessed using SMU Keithley 2400 (Cleveland, OH, USA) with a 1.5 G sunlight stimulator as a light source. Finally, an incident photon to current conversion efficiency (IPCE) test was performed using a monochromatic set-up in conjunction with an Xe-lamp (Newport, Shah Alam, Malaysia).

## 3. Results and Discussion

**Figure 1:**
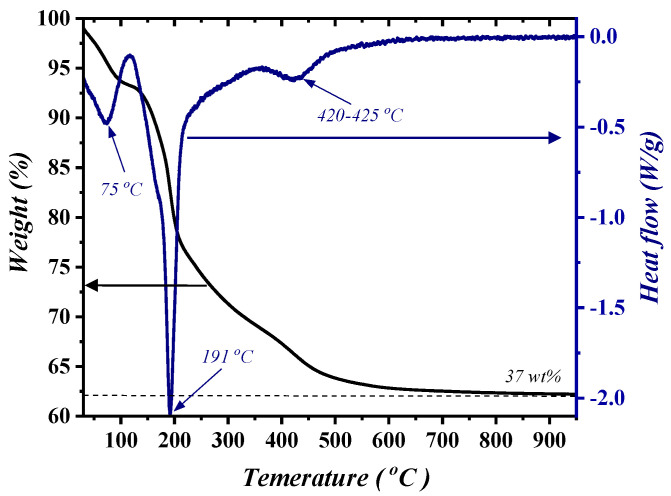
TGA/DTG profile of TiO_2_@LDH.

The thermal behavior of TiO_2_@LDH through the TGA/DTG technique is elucidated in Figure 1, which revealed the three foremost stages of weight loss with an overall degradation profile of ∆m≈37%. Specifically, the loss in $∆m_{1}$, at around 100–150 °C, is mainly because of the release of the bound water as well as moisture disappearance; this in turn was substantiated with the peak of the DTG profile at 75 °C. Instinctively, a significant weight loss ($∆m_{2}\approx 23\%)$ was perceived, ranging between 200 °C and 500 °C, which is due to the structural collapse of the LDH interlayer as well as MMO formation [29]; this was found to be in good accordance with the DTG peak at 191 °C. Thermal putrefaction, attained at 500 °C and beyond, is attributed to the CO_2_ release and possible recrystallization of TiO_2_ nanoparticles [24]. 

**Figure 2:**
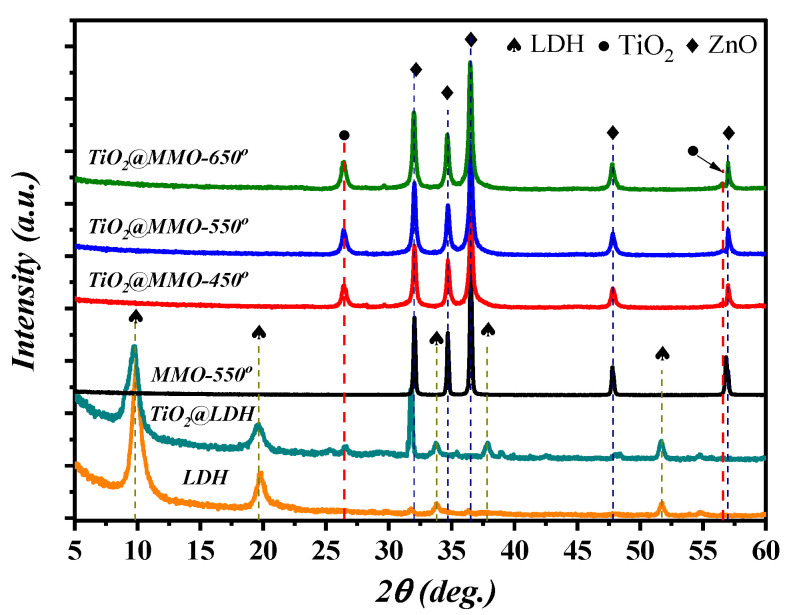
XRD patterns of pristine LDH, TiO_2_@LDH, MMO-450°, and TiO_2_@MMO-T.

The XRD patterns of the deposited layers are demonstrated in Figure 2. Pristine LDH revealed the occurrence of three distinguished peaks at 2θ = 9.9°, 19.8°, 33.8°, 37.86°, and 51.7°, which correspond to the basal planes (003) and (006) and the non-basal planes (101), (104), and (110), respectively; Additionally, the peaks attained between 30° and 37° are attributed to the formation of ZnO nanoparticles within the LDH matrix [30,31]. The XRD patterns of TiO_2_@LDH exhibited similar behavior to that of pristine LDH, with one additional peak at around 26.5° due to the existence of the TiO_2_ (101) plane [30]. The post-processing treatment (annealing) of TiO_2_@LDH at 550 °C resulted in the collapse of LDH geometry and MMO peaks formation. These peaks correspond to the crystal formation of ZnO in accordance with PDF 89-1397; in particular, the peaks obtained at around 32°, 34.6°, and 36.5° correspond to planes of (100), (002), and (101). Moreover, patterns of TiO_2_@MMO-T revealed the higher crystallinity of TiO_2_ compared to that attained in the TiO_2_@LDH spectrum (Table 1); this could be due to the effect of post-processing treatment at relatively high temperatures. It was proven that FWHM, obtained using XRD analysis, can be utilized as a crystal quality indicator, wherein FWHM is inversely proportional to the crystallite size [32]. Herein, the results presented in Table 1, in accordance with the (100) plane of ZnO hexagonal structure, indicate a higher crystal quality at relatively higher post-processing temperatures. Such a singularity was not clearly observed in the TiO_2_ profile.

**Figure 3:**
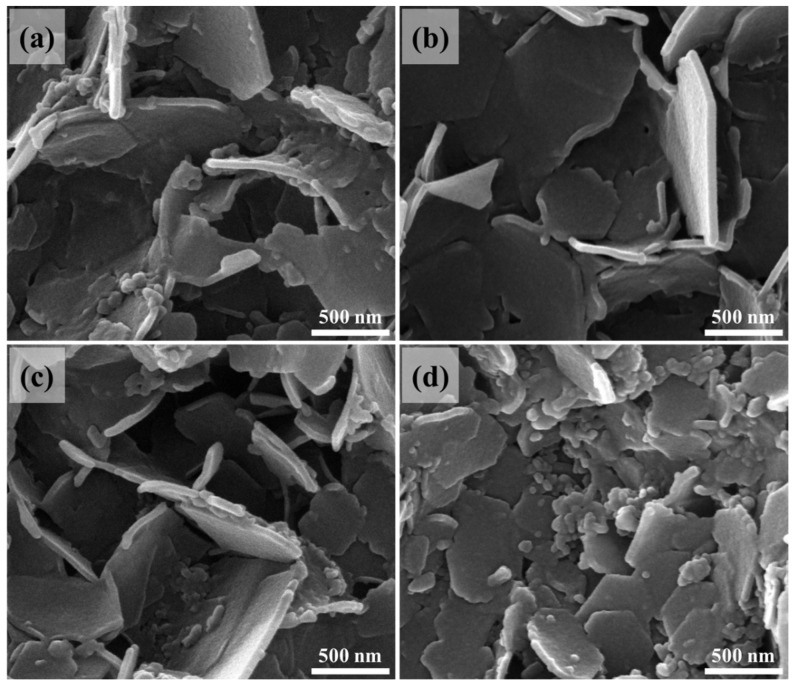
Topographies of the deposited layers (**a**) MMO, (**b**) TiO_2_@MMO-450°, (**c**) TiO_2_@MMO-550°, and (**d**) TiO_2_@MMO-650°.

The FE-SEM features of the fabricated layers are illustrated in Figure 3a–d, through which the topographies, in general, indicated the occurrence of an upright arrangement sheet-like morphology; this can be clearly observed at a temperature of 450° (Figure 3b). Moreover, the attained morphology showed a slight distortion at higher post-processing temperatures, such as temperatures of 550° and above (Figure 3a,c,d). The thickness of the attained sheet-like formation was found to be 48.7 nm, 52.75 nm, 43.11, and 38.71 nm, respectively, for the MMO prepared at 550° as well as TiO_2_-MMO-T, where T represents the post-processing temperatures (450°, 550°, and 650°).

**Figure 4:**
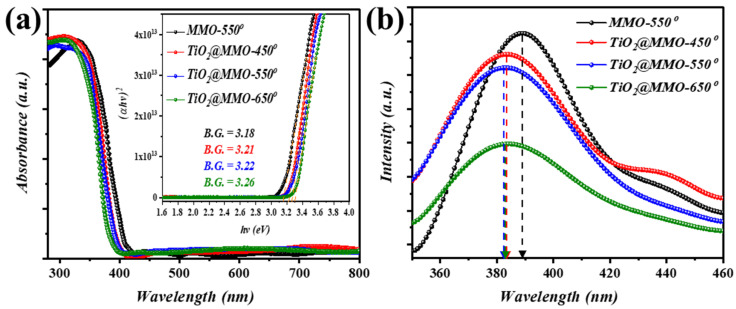
Optical characteristics of the deposited MMO-450° and TiO_2_@MMO-T layers; (**a**) UV–Vis and (**b**) PL.

Figure 4 depicts the optical performance of the attained MMO-550 as well as the TiO_2_@MMO-T films. A clear cut-off phenomenon was noticed at around 390 nm, concerning the optical behavior of ZnO attained within the MMO matrix (Figure 4a). The addition of TiO_2_ within the MMO matrix resulted in a hypso-chromic shift towards lower wavelength (TiO_2_@MMO-450°). Interestingly, the post-processing temperature resulted in a deeper hypso-chromic shift; this in turn can be verified via the bandgap investigation (inset into Figure 4a) which was estimated using Tauc relation [33,34]. A noticeable alteration in the attained optical band gap was noticed as the post-processing temperature progressed. The attained optical phenomenon was also investigated using PL spectroscopy (Figure 4b). A similar trend was noticed, where higher post-processing temperatures showed blue-shift behavior with respect to the optical bend gap values obtained. 

**Figure 5:**
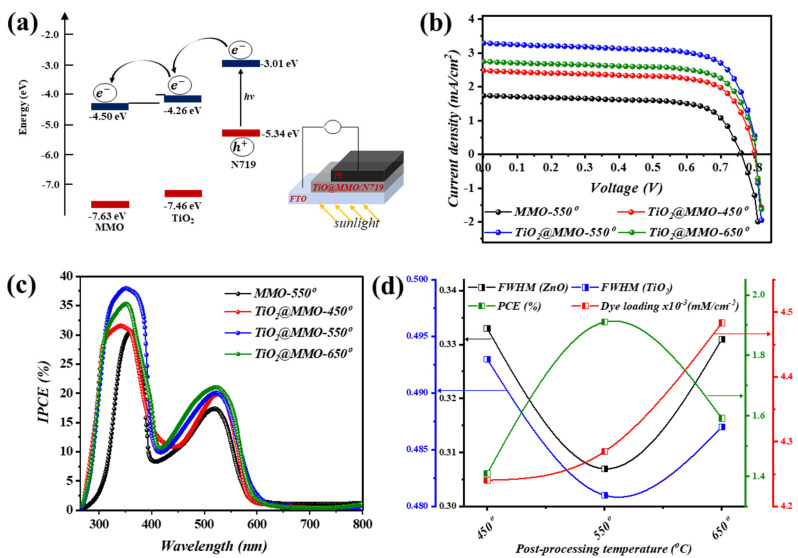
(**a**) band diagram geometry, (**b**) J–V characteristics, and (**c**) IPCE of the fabricated DSSCs, while (**d**) demonstrates a variation in FWHM of ZnO and TiO_2_ phases, PCE, and dye loading amount.

Figure 5a illustrates the band diagram geometry of the proposed work, wherein stimulated sunlight is absorbed via N719 as the main absorption layer and consequently excited electron/s, from HOMO to LUMO, are injected within the semiconductor layer, leading to current circulation [24]. The current density–voltage (J–V) characteristics of the proposed MMO-450° and TiO_2_@MMO-T DSSC devices are shown in Figure 5b, where T represents the post-processing temperature. As presented in Table 2, the fabricated devices exhibited a short circuit current ranging from 1.74 to 3.29 (mA/cm^−2^), while an almost similar open circuit voltage of ~0.80 (V) was observed for all devices. Synchronously, the highest power conversion efficiency (PCE) was noticed, with the TiO_2_@MMO layer post-processed at 550°, with a value of 1.91%. This indicates an enhancement of 109.9% compared to MMO-550° DSSC (0.91%), which in turn reveals the active role of TiO_2_ in boosting the electron transportation efficiency within the MMO matrix [35]. The latter could be explained through a superior surface area, which successively allows higher dye N719 absorption through the surface for TiO_2_@MMO-450° and TiO_2_@MMO-550°. The attained J–V outcomes are in an uptight agreement with the XRD investigation and the FWHM profile, as shown in Table 1; a higher crystal quality could lead to easier electron mobility through which faster electron transfer is attained [36]. The dye loading amount (Figure 5d) reveals a relatively up-right agreement with devices attained at 450 °C and 550 °C; however, such an observation was not noticed for DSSC obtained at 650 °C; further details are presented in the Supplementary Materials. Figure 5c depicts the incident photon-to-current efficiency (IPCE) profile of the fabricated DSSC devices in which two foremost peaks were observed at around 375 nm and 530 nm. The former can be attributed to the MMO and TiO_2_@MMO self-excitation within the UV spectrum, while the latter is chiefly caused by incident photons absorbed through dye N719 in the fabricated geometry. The quench in the IPCE curves at around 450 nm and the wavelengths above 600 nm suggest the low absorption of incident photons by the utilized dye N719. The proposed system, concerning the utilization of metal doped MMO, demonstrated a comparable overall efficiency in comparison to similar geometries (Table 3). It is worth mentioning that the demonstrated IPCE revealed slightly different peak positions compared to that attained during UV–Vis analysis, which could be due to the presence of dye N719 within the latter arrangement.

## 4. Conclusions

A series of sheet-like TiO_2_@Zn/Al-MMO was efficaciously prepared via co-precipitation and hydrothermal techniques, in which TiO_2_@Zn/Al-LDH was utilized as a precursor. Simultaneously, the attained TiO_2_@Zn/Al-MMO sheets were used for DSSC application by means of post-treatment. The microstructural and optical characteristics of the fabricated sheets were systematically elaborated. Specifically, a sheet-like thickness of 43.11 nm delivered a considerable dye loading value of 4.28 $\times 10^{-3}$ mM/cm^−2^ for the optimum device (TiO_2_@MMO-550°), which in turn lead to a relatively efficient overall PCE. In detail, the optimum device exhibited J_sc_ and V_oc_ values of 0.81 V and 3.29 mA/cm^2^, respectively; the overall PEC was found to be 1.91%. This in turn resulted in a 35.6% enhancement for DSSC fabricated at a post-processing temperature of 550° compared to that attained at 450°. 

## Figures and Tables

**Table 1:** XRD characteristics of the deposited films.

Material		2θ (deg.)	FWHM (deg.)	Crystallite Size (nm)
ZnO (100)	MMO-550°	32.05°	0.201	41.29
	TiO_2_@MMO-450°	32.05°	0.333	24.89
	TiO_2_@MMO-550°	32.10°	0.307	26.95
	TiO_2_@MMO-650°	32.08°	0.331	25.04
TiO_2_ (101)	TiO_2_@MMO-450°	26.48°	0.493	16.59
	TiO_2_@MMO-550°	26.35°	0.481	17.00
	TiO_2_@MMO-650°	26.51°	0.487	16.81

**Table 2:** In-depth photovoltaic and surface features of the fabricated DSSCs.

Sample	J_sc_ (mA/cm^−2^)	V_oc_ (V)	FF	PCE (%)	Dye Load (mM/cm^−2^)
MMO-550°	1.74	0.76	0.69	0.91	$3.71\times 10^{-3}$
TiO_2_@MMO-450°	2.47	0.80	0.71	1.41	$4.24\times 10^{-3}$
TiO_2_@MMO-550°	3.29	0.81	0.72	1.91	$4.28\times 10^{-3}$
TiO_2_@MMO-650°	2.74	0.80	0.73	1.59	$4.48\times 10^{-3}$

**Table 3:** Parameters attained in the proposed work compared to other reports in similar geometries.

Materials	J_sc_ (mA/cm^−2^)	V_oc_ (V)	FF	PCE (%)	Ref.
TiO_2_@MMO	3.29	0.81	0.72	1.91	This study
TiO_2_@MMO	2.63	0.81	0.70	1.50	[24]
CuO@MMO	3.42	0.67	0.55	1.24	[25]
CuO@MMO	7.21	0.46	0.45	1.50	[26]
G@MMO	0.36	3.62	0.39	0.51	[27]
GO@MMO	0.37	4.46	0.34	0.44	[28]

## Data Availability

The data presented in this study are available on request from the corresponding author due to research further enhancement.

## Supplementary Materials

The following supporting information can be downloaded at: https://www.mdpi.com/article/10.3390/nano14110920/s1, Figure S1: Regression equation; Table S1: Variables of regression equation.
