# Supplementary material for: Fabrication and Enhanced Performance Evaluation of TiO2@Zn/Al-LDH for DSSC Application: The Influence of Post-Processing Temperature
Source: Nanomaterials (Basel). 2024 May 24;14(11):920. doi: 10.3390/nano14110920 (PMC11173877; doi:10.3390/nano14110920)
Supplement: Supplementary file 1 [file nanomaterials-14-00920-s001.zip › nanomaterials-2989012-supplementary.pdf]

nanomaterials

Supplementary Materials

Fabrication and Enhanced Performance Evaluation of TiO_2@Zn/Al-LDH for DSSC Application: The Influence of Post-Processing Temperature

Altaf Hussain Rajpar [1], Mohamed Bashir Ali Bashir [1,*], Ethar Yahya Salih [2] and Emad M. Ahmed [3]

[1] Department of Mechanical Engineering, College of Engineering, Jouf University, Sakaka 72388, Saudi Arabia; ahrajpar@ju.edu.sa
[2] Department of Renewable Energy, College of Renewable Energy and Environmental Sciences, Al-Karkh University of Science, Baghdad 10081, Iraq; ethar988@gmail.com
[3] Department of Electrical Engineering, College of Engineering, Jouf University, Sakaka 72388, Saudi Arabia; emamahmoud@ju.edu.sa
* Correspondence: mbashir@ju.edu.sa

Table S1. Variables of regression equation.

Equation	$y = a + b \times x$
R^2	0.99142
Intercept (a)	-0.3986
Slope (b)	4.7765

Dye N719 was dissolved in ethanol at different concentrations (0.1, 0.2, 0.3, 0.4 and 0.5 mM) starting with the employed dye concentration (0.5 mM) and subsequently reduced. The UV–Vis absorbance spectra were measured for the prepared dye concentrations and λ max values were recorded (Figure 5, c). Afterwards, a regression equation was created and the resultant equation is as follows:

$$y = 4.7765x - 0.3986 \quad \text{(S1)}$$

where y represents the λ max of the absorbance spectra and x is the dye concentration. Following similar procedures, the value of an unknown dye concentration loaded onto a semiconductor layer (x) can be evaluated, where the value of y is obtained from the UV–Vis measurements of any photo-anode employed.

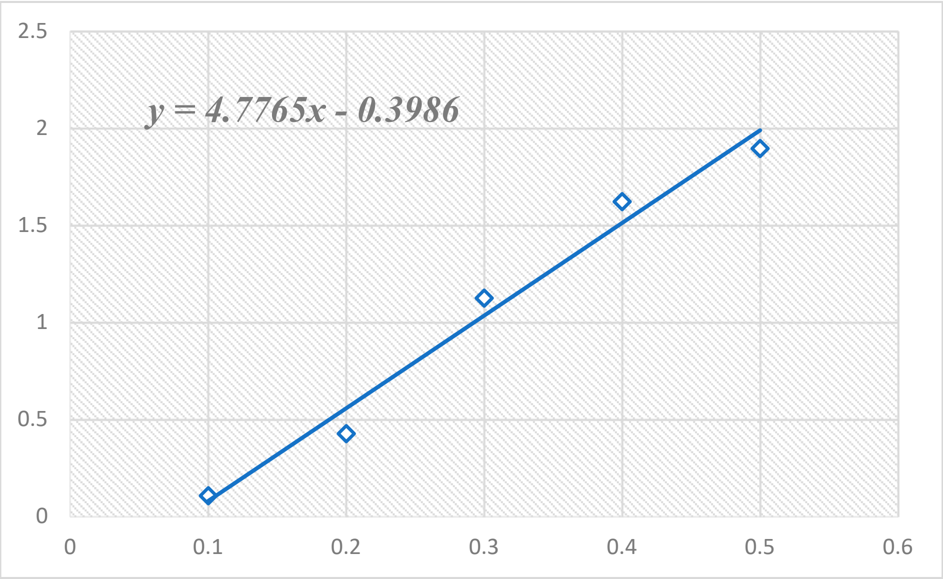

Figure S1. Regression equation.
